# Soil water storage capacity under chronosequence of revegetation in Yanhe watershed on the Loess Plateau, China

**DOI:** 10.1186/2193-1801-2-S1-S15

**Published:** 2013-12-11

**Authors:** Feng Jiao, Zhong-Ming Wen, Shao-Shan An

**Affiliations:** Institute of Soil and Water Conservation, Northwest A&F University, Yangling, Shaanxi China; Institute of Soil and Water Conservation, Chinese Academy of Science and Ministry of Water Resource, Yangling, Shaanxi China

**Keywords:** land use, Loess Plateau, soil water storage, vegetation restoration

## Abstract

The relationship between vegetation and soil moisture deserves attention due to its scientific importance and practical applications. However, the effects of soil moisture on vegetation development and succession are poorly documented. Here we study soil water storage in Yanhe watershed at northern Shaanxi on five different land uses, namely shrubland, farmland, natural grassland, woodland, and artificial grassland, and in soil under restoration for 5, 10, 15, 20 and 25. The results show that (1) soil water in soil 0-60 cm below ground is the highest in farmland, and lower in shrubland, artificial grassland, natural grassland and woodland; (2) soil water in artificial grassland and woodland decreases rapidly as the soil depth increases; whereas soil water in farmland, natural grassland, shrubland and woodland decreases; (3) soil water storage of farmland is greater than that of shrubland, artificial grassland, natural grassland and woodland; and (4) the vegetation succession in soil undergo restoration for different years on eroded soil results in a decrease in soil water storage.

## Introduction

Soil water is one of the most important resources for sustaining vegetation growth in the semiarid area of the Loess Plateau in northwestern China [1, 2]. Soil of the Loess Plateau develops from thick loess material with high water storage capacity [3, 4] of about 200-300 mm m^-1^ on average [5], so the soil water in the area functions as a reservoir regulating vegetation growth. This is the main reason why the ecosystem in this semiarid area has been sustained for thousands of years. However, evapotranspiration of many planted grasses of leguminous species, shrubs, and forests is beyond what precipitation can recharge [6–14], which restrains further growth of much planted vegetation [15].

Many studies on physiological plant ecology have been conducted focusing on the relationship between vegetation and soil water [16–22], but many aspects of vegetation and soil water remains unclear. The objective of the present study is to identify changes in soil moisture in five different land uses including shrubland, natural grassland, artificial grassland, farmland, and woodland, and changes in soil moisture and water storage after different restoration periods of plantations. The most popular natural grassland in the study area with vegetative chronosequence is also investigated to evaluate soil water storage on lands with different restoration times.

## Materials and methods

### Study area

The study area is located in the Yanhe watershed of the Loess Plateau at N36°23′-37°17′ and E108°45′-110°28′ in northern Shaanxi Province. An on-site ecology station has comprehensively managed this area for 25 years. The area is 287 km in length, and 7687 km2 in size. Ninety percent of the land is hilly, 3% is villages, rivers, and lakes, and only 7% is suitable for intensive agriculture. The study area has a sub-arid climate characterized by heavy seasonal rainfall with periodic local flooding and drought; the average annual rainfall at the experimental site is 497 mm (1970-2000, CV22%) with distinct wet and dry seasons. The rainy season starts in July and ends in October, and rainfall in August accounts for 23% of the annual precipitation. The annual reference evapotranspiration is approximately 1,000 mm. Most of the lands are located at 900-1500 m altitude, closely dissected, and sharp-edged with very steep slopes (the slopes are 40%). The topography, soil type, soil and land-use patterns of Yanhe watershed are very typical in the Loess Plateau. Land-use types include sloping land, terraces, orchards, woodland, shrubland, natural grassland, wasteland and others [23].

### Study approach and sampling design

The chronosequence method was used because of a conversion history in this area. The management was similar to previous practices according to known cultivation climate, topography, and soil type. Soil samples were collected in August 2006. The soil samples were taken with a corer and dried at 105°C for 12 h. Soil moisture was measured using the oven-drying method (Nanjing Institute of Soil Research, Chinese Academy of Sciences, 1980). Soil samples were taken at 10 different depths: 20, 40, 60, 80, 100, 120, 140, 160, 180 and 200 cm below ground with 5 replicates per vegetation community. At each location, the soil bulk densities of samples taken from 0-20, 20-40 and 40-60 cm below ground were measured using a 100-cm^3^ cylinder, respectively.

Soil rehabilitation in relation to vegetative cover is commonly studied by monitoring changes in plants and soil along a vegetative chronosequence developed on similar soil under similar climates [16]. This chronological approach has been widely used in applied ecosystem researches [24] and is considered retrospective, because existing conditions are compared with known original conditions and treatments. Retrospective approach was used in this study because of the availability of adjacent vegetation communities established 5, 10, 15, 20 and 25 years ago on eroded soils with similar properties. These vegetation communities provided a time gradient of grass occupancy on similar sites. Changes in soil properties were measured by comparing sites of different ages. Five age series (5-, 10-, 15-, 20- and 25-year-old vegetation community) were found adjacent to the study area, and have undergone light livestock grazing in recent years. Within each community (5, 10, 15, 20 and 25-year-old vegetation), five sampling sites were selected and samples were collected with five replicates. Also, five non-vegetated lands near the planted sites (farmland) were chosen as controls for the chronosequence.

### Data analysis

Soil water storage (soil water quantity within a certain depth) was calculated based on soil moisture and soil bulk density. Soil water storage has been used as the key factor to evaluate soil moisture, because calculations based on soil volume eliminated the influence of soil depth [25].

Soil moisture analysis of variance (ANOVA) and correlation were carried out using the SPSS11.0 procedures for sites in different succession stages. Duncan's test (p < 0.05) was used to compare means of soil variables when the results of ANOVA were significant at p < 0.05.

### Calculation of soil water storage

Soil water storage (soil water quantity within a certain depth) was calculated based on soil moisture and soil bulk density. Soil water storage has been used as the key factor to evaluate soil moisture, because calculations based on soil volume eliminated the influence of soil depth (Hu, 1992). Soil water storage [(mm)] for a given soil layer  was calculated using the following formula:1

Where  is soil moisture (%),  is soil bulk density (g cm-3), and  is the depth of the layer  (cm).

In this research, soil water storage at 0-60 cm, 60-120 cm and 120-200 cm below ground were calculated according to formula (1). Total soil water storage at 0 to 200 cm below ground was calculated as the sum of the soil water storage at 0-60 cm, 60-120 cm and 120-200 cm below ground.

## Results and discussions

### Soil moisture in different land uses

In the surface layer (0-60 cm), soil moisture was the highest in farmland, followed by shrubland, artificial grassland, natural grassland and woodland (Table 1). Compared with that in farmland, soil moisture decreased in shrubland, artificial grassland, natural grassland and woodland by 18.50, 21.53, 24.38, and 31.44%, respectively. Same as in the surface layer, in the 60-120 cm layer, the soil moisture was the highest in farmland, followed by shrubland, artificial grassland, natural grassland and woodland. Soil moisture in the 60-120 cm layer in farmland, shrubland and artificial grassland were similar to that in the surface layer in these land uses. However, soil moisture in the 60-120 cm layer decreased in natural grassland and woodland by 5.83 and 16.64%, respectively, when compared with that in the surface layer in these land uses (Table 1).

**Table 1:** Soil moisture and bulk density in different land-use patterns

Item	Soil layer		Farmland	Artificial grassland	Natural grassland	Woodland	Shrubland
	0-60 (cm)	Mean (%)	9.03 ± 2.68	7.43 ± 2.50	7.26 ± 2.03	6.87 ± 1.93	7.62 ± 3.22
		CV	0.30	0.34	0.28	0.28	0.42
Land use	60-120 (cm)	Mean (%)	8.99 ± 1.82	7.23 ± 2.23	6.86 ± 3.06	5.89 ± 1.44	7.63 ± 2.60
		CV	0.20	0.31	0.45	0.24	0.34
	120-200 (cm)	Mean (%)	8.97 ± 1.46	6.00 ± 1.86	7.00 ± 3.34	5.41 ± 1.71	7.13 ± 2.24
		CV	0.16	0.31	0.48	0.32	0.31
	0-20 (cm)	Mean (g·cm^-3^)	1.28 ± 0.06	1.24 ± 0.03	1.32 ± 0.07	1.35 ± 0.07	1.27 ± 0.11
		CV	0.05	0.02	0.05	0.05	0.09
Bulk density	20-40 (cm)	Mean (g·cm^-3^)	1.35 ± 0.05	1.34 ± 0.09	1.32 ± 0.03	1.37 ± 0.07	1.34 ± 0.07
		CV	0.04	0.07	0.03	0.05	0.05
	40-60 (cm)	Mean (g·cm^-3^)	1.32 ± 0.09	1.46 ± 0.08	1.36 ± 0.08	1.37 ± 0.05	1.36 ± 0.04
		CV	0.07	0.06	0.06	0.04	0.03

In the 120-200 cm layer, soil moisture was the highest in farmland, followed by shrubland, natural grassland, artificial grassland and woodland. Soil moisture in natural grassland and shrubland was similar, and soil moisture in artificial grassland and woodland was almost the same, which was lower than that in other land uses (Table 1). Soil moisture in the 120-200 cm layer in farmland, shrubland and natural grassland were similar to that in the surface layer in these land uses. However, soil moisture in the 120-200 cm layer decreased in artificial grassland and woodland by 23.83 and 26.99%, respectively, when compared with that in the surface layer in these land uses (Table 1).

### Soil water storage in different land uses

In the surface and the 60-120 cm layer, water storage in farmland soil was higher than that in other land uses (Table 2). Although some data obtained from farmland fluctuated because of farming or soil preparation. The overall soil water storage in farmland was higher than that in other land uses. Artificial grassland and shrubland soils had almost the same soil water storage. Natural grassland and woodland had low soil water storage. In the 120-200 cm layer, water storage in farmland soil was higher than that in other land uses (Table 2). Soil water storage in natural grassland was almost the same as that in shrubland. Woodland had the lowest soil water storage (Table 2). Similarly, in the 0-200 cm layer, farmland had the highest soil water storage, followed by shrubland and woodland (Table 2).

**Table 2:** Soil water storage (mm) under different land-use patterns (%)

Land use	Samples	0-60 (cm)	60-120 (cm)	120-200 (cm)	0-200 (cm)
Farmland	5	71.11 ± 21.01	70.93 ± 14.58	94.16 ± 13.69	236.21 ± 47.58
Artificial grassland	3	60.70 ± 22.21	63.88 ± 21.66	69.51 ± 18.73	194.09 ± 40.27
Natural grassland	7	58.49 ± 17.02	56.54 ± 27.07	76.58 ± 38.76	191.61 ± 78.93
Woodland	7	56.53 ± 17.50	48.38 ± 11.81	59.39 ± 19.11	164.30 ± 25.32
Shrubland	5	60.69 ± 26.79	62.55 ± 22.09	77.90 ± 25.48	201.14 ± 56.20

### Moisture and water storage in soil with different restoration time

Five replicated soil samples were collected from six sites with the same restoration time of 5, 10, 15, 20 or 25 years, respectively. Also, five nonvegetated lands in the vicinity of the planted sites (farmland) were chosen as controls for the chronosequence. It was assumed that soil in sites with 5-, 10-, 15-, 20- or 25-years of restoration was initially similar to the farmland site (Table 3). After 5, 10, 15, 20 and 25 years of natural grass occupancy, vegetation succession resulted in a fluctuation of soil moisture in the eroded soils (Table 3). Soil moisture in the surface layer of farmland was 9.89%. Soil moisture in 5-, 10-, 15-, 20-year-old grassland in the surface layer were 8.20%, 9.60%, 7.05%, 7.84%, and 8.65%, respectively. Soil water storage in the surface layer of farmland was 77.84 mm. Soil water storage in 5-, 10-, 15-, 20-year-old grassland at the surface layer were 44.52 mm, 78.19 mm, 56.41 mm, 68.81 mm, and 69.47 mm, respectively (Table 4).

**Table 3:** Moisture and bulk density of farmland soil with different restoration periods

Restoration (years)	Soil moisture (%)			Bulk density (g·cm^-3^)		
	**0-60 (cm)**	**60-120 (cm)**	**120-200 (cm)**	**0-20 (cm)**	**20-40 (cm)**	**40-60 (cm)**
25	8.65 ± 2.61^abcd^	7.78 ± 3.07^abcd^	7.03 ± 1.97^bcd^	1.26 ± 0.08^c^	1.38 ± 0.14^ab^	1.38 ± 0.11^ab^
20	7.84 ± 3.14^abcd^	7.65 ± 2.93^abcd^	7.90 ± 1.82^abcd^	1.31 ± 0.08^abc^	1.39 ± 0.10^ab^	1.40 ± 0.06^a^
15	7.05 ± 1.31^bcd^	6.94 ± 1.60^cd^	7.24 ± 2.48^bcd^	1.30 ± 0.06^bc^	1.32 ± 0.07^abc^	1.37 ± 0.05^ab^
10	9.60 ± 1.56^ab^	8.74 ± 0.99^abcd^	6.47 ± 0.71^d^	1.31 ± 0.09^abc^	1.37 ± 0.06^ab^	1.39 ± 0.04^ab^
5	8.20 ± 2.28^abcd^	8.12 ± 2.38^abcd^	9.00 ± 1.83^abcd^	1.33 ± 0.04^abc^	1.40 ± 0.04^ab^	1.38 ± 0.04^ab^
0	9.89 ± 1.83^a^	9.60 ± 1.17^ab^	9.41 ± 1.12^abc^	1.27 ± 0.06^c^	1.35 ± 0.05^abc^	1.31 ± 0.10^abc^

^abcd^: significant differences between sites based on LSD (p < 0.05).

**Table 4:** Soil water storage (mm) of farmland soil with different restoration periods

Restoration (years)	Samples	0-60 (cm)	60-120 (cm)	120-200 (cm)	0-200 (cm)
25	5	69.47 ± 22.34^bc^	64.18 ± 24.58^cd^	77.77 ± 22.38^abc^	211.43 ± 49.56^a^
20	5	64.81 ± 27.40^bcd^	64.79 ± 26.27^bcd^	88.93 ± 22.42^bcd^	218.53 ± 75.81^a^
15	5	56.41 ± 11.53^cd^	56.94 ± 13.33^cd^	78.90 ± 26.72^abc^	192.26 ± 36.45^a^
10	5	78.19 ± 12.62^abc^	72.60 ± 7.48^bc^	71.72 ± 8.23^bc^	222.51 ± 15.09^a^
5	5	44.52 ± 12.71^d^	67.58 ± 21.52^bcd^	99.68 ± 21.59^a^	211.79 ± 53.83^a^
0	5	77.84 ± 14.55^abc^	75.55 ± 10.28^abc^	98.39 ± 10.02^a^	251.78 ± 31.78^a^

^abcd^: significant differences between sites based on LSD (p < 0.05).

## Conclusion

Land use has an obvious effect on soil moisture and soil water storage. Farmland has higher soil moisture than other land uses, although the latter are located on the slope rather than intra-gully alluvial land, which is mostly used as forestlands or orchards. In most cases, table land has sufficient levels of soil moisture, but after long period of cultivation, the land degrades year by year. Our results indicate that the establishment and development of vegetation succession on eroded soil result in significant degenerations of soil moisture and temporary soil water storage. With increased plantation age, it is possible to recover soil moisture and soil water storage to a certain degree.
